# A Computational Fluid Dynamic (CFD) Simulation of PM_10_ Dispersion Caused by Rail Transit Construction Activity: A Real Urban Street Canyon Model

**DOI:** 10.3390/ijerph15030482

**Published:** 2018-03-09

**Authors:** Yang Wang, Ying Zhou, Jian Zuo, Raufdeen Rameezdeen

**Affiliations:** 1 School of Civil Engineering, Wuhan University, Wuhan 430072, China; zoe0718@foxmail.com; 2 School of Architecture & Built Environment, Entrepreneurship, Commercialization and Innovation Centre (ECIC), The University of Adelaide, Adelaide, SA 5005, Australia; jian.zuo@adelaide.edu.au; 3 School of Natural and Built Environments, University of South Australia; Adelaide, SA 5001, Australia; Rameez.Rameezdeen@unisa.edu.au

**Keywords:** urban street canyon, PM_10_, construction activity, computational fluid dynamic (CFD), height ratio, wind direction

## Abstract

Particle emissions derived from construction activities have a significant impact on the local air quality, while the canyon effect with reduced natural ventilation contributes to the highest particulate pollution in urban environments. This study attempted to examine the effect of PM_10_ emissions derived from the construction of a rail transit system in an urban street canyon. Using a 3D computational fluid dynamic (CFD) model based on a real street canyon with different height ratios, this study formulates the impact of height ratio and wind directions on the dispersion and concentration of PM_10_. The results indicate that parallel flow would cause the concentration of PM_10_ at the end of the street canyons in all height ratios, and the trends in horizontal, vertical and lateral planes in all street canyons are similar. While in the condition of perpendicular flow, double-eddy circulations occur and lead to the concentration of PM_10_ in the middle part of the street canyon and leeward of backwind buildings in all height ratios. Furthermore, perpendicular flow will cause the concentration of PM_10_ to increase if the upwind buildings are higher than the backwind ones. This study also shows that the dispersion of PM_10_ is strongly associated with wind direction in and the height ratios of the street canyons. Certain measures could, therefore, be taken to prevent the impact on people in terms of the PM_10_ concentration and the heights of street canyons identified in this research. Potential mitigation strategies are suggested, include measurements below 4 m according to governmental regulations, dust shields, and atomized water.

## 1. Introduction

With declining urban environment quality due to increasing air pollution levels, the study of pollutant transport in urban areas has attracted considerable interest in the past few decades [1,2]. The concentration of ultrafine particles has largely been singled out for their impact on human health, urban climate change and visibility impairment [3,4,5,6,7,8]. Ultrafine particles originate mainly from road dust, construction works, vehicle traffic and fuel oil combustion [9,10,11]. Due to rapid urbanization and the resultant development, particles from construction activities play a crucial role in determining urban air quality [12,13].

A street canyon has been defined as the relatively narrow strip in-between buildings along both sides of a road [3]. With the increase of high-rise buildings that are densely packed along narrow streets, there is a high tendency for pollutants to accumulate as there is not enough room for their dispersion [14,15]. The pollution level is general high in street canyons, which exposes local residents to health threats [16]. Because of the adverse health effects of these particles, evaluating their concentration and dispersion in urban street canyons becomes a very important research focus.

Construction activities pose substantial environmental impacts and air-quality issues. As rail transit systems are often located along street canyons, the earthworks associated with their construction generates an enormous amount of sustained particles that linger for a long time. PM_10_ mass concentrations are mainly associated with coarse mineral dust particles, which is one of the essential indicators of air quality. Past studies have shown that in the vicinity of construction sites, PM_10_ emissions can easily exceed the allowable limit. Moreover, dominated by PM_1_/PM_10_ and PM_2.5_/PM_10_ mass ratios, the particle-size distribution around construction sites can have significant detrimental effects [17]. 

Many numerical models are available to evaluate the particle-dispersion principles in street canyons from different perspectives: namely, the street canyon geometry, aspect ratio (AR), wind direction, wind speed etc. Similarly, others have investigated the emission factors and their impacts on air quality surrounding construction activities. For example, the OSPM model (operational street pollution model) takes into account the geometric details of buildings located in the street canyon and the gaps between these buildings. However, only very few researchers have studied the characteristics of real-world emissions of construction activities [18]. Such real street canyon modeling could generate information often ignored in most other studies, such as the influence of the heights of adjacent buildings (height ratio) and gaps between buildings which are very useful for devising mitigation strategies.

This paper attempts to fill in this research gap. The focus is placed on PM_10_ dispersion and flow structure by using the computational fluid dynamics (CFD) method and examines the influences of canyon configuration of height ratio and wind direction based on real observations conducted in Wuhan, China. The main objective of this work is to identify the scope and influence of PM_10_ from heavy construction activities for different street configurations and meteorological conditions. The findings can assist construction contractors to develop prevention strategies for PM_10_ pollution and improve air quality within and outside construction sites.

## 2. Literature Review

### 2.1. Method of Analysis

Several methods and technical tools have been employed in previous studies to examine flow structure and particle dispersion in the context of urban street canyons. Some common tools and methods are: wind-tunnel experiments, numerical simulations, field measurements, etc. [19,20,21,22,23]. 

Using a pollutant analyzer, field measurements are widely applied in urban street canyon studies to obtain the real-time data about wind flow and particle concentrations at specific locations. Faber, Drewnick and Borrmann [17] used online instruments to examine the characteristics of particles emitted by earthworks and road work so as to reveal the emission factor for PM_10_ and formulate the predominant mechanical processes that emit dust particles. They concluded that PM_10_ emissions from construction activities often exceed the threshold set by the European standard for PM_10_. Kumar, Fennell and Britter [24] measured the particles in UK street canyons by the “fast response differential mobility spectrometer” to investigate particle distribution, concentration and vertical variations according to wind direction. Their results also showed the distribution of particle numbers varied depending on the side of canyons [25]. Li, Wang, Tu, Liu and Huang [23] investigated the change in vertical variation of particle-size distribution. Therefore, field measurements can offer an enormous amount of useful information on the flow structures and particle-concentration distribution within street canyons. However, there are a number of limitations associated with field measurements such as “low spatial resolution, uncontrollable meteorological conditions and complex building configurations” [3].

To solve these problems, laboratory-scale physical modeling approaches, such as wind-tunnel experiments, are widely used as the “fully controllable upwind boundary conditions for airflow and canyon geometries” [3]. Sensors can be used to detect the flow dynamics of particles in a street canyon [26]. Similarly, wind-tunnel measurements have often been conducted with canyon geometries to model the transport patterns of air, heat and particles across various scales of urban areas [21,27,28]. However, there are limitations associated with these approaches such as low spatial resolution, high cost, and uncontrollable meteorological conditions [3].

CFD have been widely employed in both academic and industry settings. As a cost-effective approach, CFD allows the investigation of pollution issues in a street canyon, such as concentration and transport, in higher spatial resolution [3]. Various models are available to investigate the dispersion of particles within different environments, such as box models and fluid dynamics models [29]. Wind flow is strongly characterized by its turbulent nature. As a result, there are common approaches to deal with this problem. Reynolds-Averaged Navier–Stokes (RANS) based turbulence models, such as the *κ*-*ε* closure scheme, are getting popular as most RANS models are established in the linear eddy viscosity assumption and are numerically stable with the least computational demand [30,31]. While facing the complicated mixed turbulence situation, it is appropriate to employ large eddy simulation (LES) and direct numerical simulation (DNS) models as they require more prohibitive mesh resolutions for small eddies [32]. 

Overall, phenomena occurring in the field may not always be captured in modeling, while a non-validated model does not provide much value, and the only way to validate it is with measurements. Therefore, these tools and methods play different significant roles, and all of them are in fact related to each other. In this research, different height ratios and wind directions in a street canyon will be simulated; in this way, the CFD method can outperform experiments in an economic and effective way by supplying comprehensive data for all relevant variables that streamline the decision-making process [19].

### 2.2. Canyon Configurations

Canyon configurations are often characterized by aspect ratio, including H/W (i.e., ratio of building height to street width) and L/W (i.e., ratio of building length to street width) [19,33]. A street canyon is asymmetrical if the building heights on both sides are unequal. Asymmetrical street canyons are divided into two types: step-up and step-down.

Recently, a number of studies have been undertaken to assess flow and pollutant dispersion in symmetric canyon models, and the aspect ratios are the most significant parameters. A street canyon with an aspect ratio of 0.5, 1.0 and 2.0 could be employed, and benchmarking the simpler cases gives confidence in the more complicated street canyon case so that the pollutant distribution and concentration characteristics could be investigated [34,35]. Similarly, field-size canyon models with various length to height ratio (L/H) and roof shapes were analyzed to obtain the characteristics of pollutant transport and diffusion [36].

While regard to their geometry, a typical city is an asymmetric street canyon. Such kinds of canyon would produce a recirculation vortex in terms of different flow structures and pollutant dispersion, depending on the adjacent building heights along the street. For the symmetrical and step-up street canyon, the pollutant concentration is considered as important factor at the leeward than the windward wall [19]. On the contrary, the concentration in step-down canyon in the windward side is slightly greater than leeward [37]. Meanwhile, the flow structure, including the main vortices and their intensity, the direction of the rotation, changes for step-up and step-down patterns, which leads to a change of pollutant accumulation accordingly [38]. 

Crossroad gaps often exist in street canyons, and semi-empirical models allow gaps between buildings [16]. However, several works of research focusing on the gaps indicates that a crossroad gap between buildings led to a significantly different wind profile inside the canyon [36]. It takes a bit longer to dilute particles during the cross canyon winds in a real street canyon model due to such a gap between buildings [36,39].

Canyon configuration, wind direction and speed have also been considered in street-canyon modeling. Perpendicular, oblique and parallel wind was found to show a different flow structure and formation of vortex. There is a complicated transition of flow structure within different types of canyon [40]. While in the condition of parallel flow to the street axis, the mean vertical velocity is almost zero [41]. A gradual decrease of pollutant concentration was identified on both sides of street along walls [19]. Furthermore, the longitudinal velocity along the canyon varies according to H/W ratios [42].

Existing studies predominately have focused on the street canyon configuration, such as the aspect ratio and height ratio, in order to examine the impact of street geometry on the flow structure, the vortexes and the pollutant dispersion [19,33,34,35,38,39]. Most of these studies are based on idealized modeling in a CFD approach, overlooking factors that exist in real street canyons, such as irregular streets and the existing gaps in adjacent buildings [34,43]. In fact, combined with the aspect ratio, height ratio and wind direction, these factors will also change the flow structure inside the street canyon [19]. Furthermore, as regards the particle dispersion in street canyon, most research has taken traffic as the example. In order to predict the main emissions source, the traffic-based emission model, such as the ADMS-Urban model, is used as emission factor, which covers the traffic factors, including the number of vehicles per hour, vehicle types and average vehicle speed [39]. While particles from construction activity are deduced from earthworks and road works, the bases of emission factors are quite different [17]. Therefore, it remains unclear what is the flow structure and how the particle disperses based on a real street canyon with irregular buildings and adjacent buildings with different height ratios as a result of construction activity. Taking the rail transit construction project as an example in this study, a three-dimensional CFD model was used to better understand the pollutant transport process and distribution pattern in a field-size canyon model. This analyzed the influences on distribution and concentration of particles in symmetrical and asymmetrical street canyons in different wind directions, and identified that the source and configuration mostly contributes to the particle concentrations via the standard *κ*-*ε* turbulence closure model.

## 3. Methodology

In this study, Ansys 14.0 was used to execute the CFD simulation. The wind flow in the street canyon is numerically stable, so the *κ*-*ε* turbulence model was used in this study. Based on turbulent kinetic energy and rate of turbulent dissipation transport equations, the flow structure will be formulated by *κ*-*ε* closure scheme. The *κ*-*ε* equation is described below [35]:

(1) $$\frac{\partial }{\partial t}\left(\rho \kappa \right)+\frac{\partial }{\partial x_{i}}\left(\rho \kappa \mu_{i}\right)=\frac{\partial }{\partial x_{j}}\left[\left(\mu +\frac{\mu_{t}}{\sigma_{\kappa }}\right)\frac{\partial \kappa }{\partial x_{j}}\right]+G_{\kappa }+G_{b}-\rho \varepsilon$$

(2) $$\frac{\partial }{\partial t}\left(\rho \epsilon \right)+\frac{\partial }{\partial x_{i}}\left(\rho \varepsilon \mu_{i}\right)=\frac{\partial }{\partial x_{j}}\left[\left(\mu +\frac{\mu_{t}}{\sigma_{\varepsilon }}\right)\frac{\partial \varepsilon }{\partial x_{j}}\right]+C_{1\varepsilon }\frac{\varepsilon }{\kappa }\left(G_{\kappa }+C_{3\varepsilon }G_{b}\right)-C_{2\epsilon }\rho \frac{\varepsilon^{2}}{\kappa }$$

In which κ is defined as kinetic energy of turbulence flow; ε as the rate of turbulent dissipation transport; ρ is the density; *t* is time; *x_i_* and *x_j_* present the Cartesian coordinate system; $\mu_{i}$ is the velocity of flow; μ is kinetic viscosity; $G_{\kappa }$ is turbulence production item; $G_{b}$ is the turbulent kinetic energy generation caused by Buoyancy; $\sigma_{\kappa }\;\mathrm{and}\;\sigma_{\varepsilon }$ are the turbulence coefficient, $\sigma_{\kappa }$ = 1.0, $\sigma_{\varepsilon }$ = 1.3; $\mu_{t}$ = $\rho C_{\mu }\frac{\kappa^{2}}{\varepsilon }$, $C_{\mu }=$ 0.09; $C_{1\varepsilon }$ = 1.44, $C_{2\varepsilon }$ = 1.92, $C_{3\varepsilon }$ = 1.44.

As there are comparatively fewer particles in the air, a discrete phase model is used. For the aerosol movement near the ground, the Euler method is used. The trajectory of particles in the street canyon is tracked by Lagrange’s method and simulated by the random walk model in the random track model. The effects of diverse forces such as Saffman, drag, and gravity can be assessed. The dynamic equations of particulate transport can be expressed as follows [35,44]:

(3) $$\frac{\pi d_{P}^{3}\rho_{P}}{6}\frac{du_{P}}{dt}=F_{drag}+F_{gravity}+F_{saffman}$$

(4) $$F_{drag}=-\frac{1}{6}\pi d_{P}^{3}\rho_{P}\frac{1}{\tau }\left(u_{P}-u\right)$$

(5) $$F_{gravity}=\frac{1}{6}\pi d_{P}^{3}\left(\rho_{P}-\rho \right)g_{i}\delta_{i}$$

(6) $$F_{saffman}=\frac{1}{6}\pi d_{P}^{3}\rho_{P}\frac{5.188\nu^{0.5}d_{ij}}{Sd_{P}{\left(d_{lk}-d_{kl}\right)}^{0.25}}\left(u_{P}-u\right)$$

where: $u_{P}$ is the particle velocity; u is the fluid velocity; $\rho_{p}$ is the particle density; ρ is the fluid density; $d_{P}$ is the particle diameter; *S* is the density ratio between a particle and adjacent fluid; ν is the kinematic viscosity; $\delta_{i}$ is the unit delta function; $g_{i}$ is the hydrodynamic viscosity; τ is the particle relaxation time; and $d_{ij}=\left(u_{ij}+u_{ji}\right)/2$ is the deformation rate tensor.

## 4. Case Study

### 4.1. Case Selection

Urban rail transit is a very popular public transport mode around the world. The construction of urban rail transit is a typical project in street canyons. While urban rail transit systems have been in operation for a long time in developed countries, they are still at infancy in developing countries. Therefore, a number of projects have been initiated in recent years to manage the ever-increasing demand for urban commuting in developing countries. For example, in China, 43 cities have commissioned urban rail transit projects reaching a total of 8600 km up to 2016 [45]. Wuhan, the capital of Hubei Province, is planning to add 1045 km of urban railway lines by year 2021 [46].

**Figure 1:**
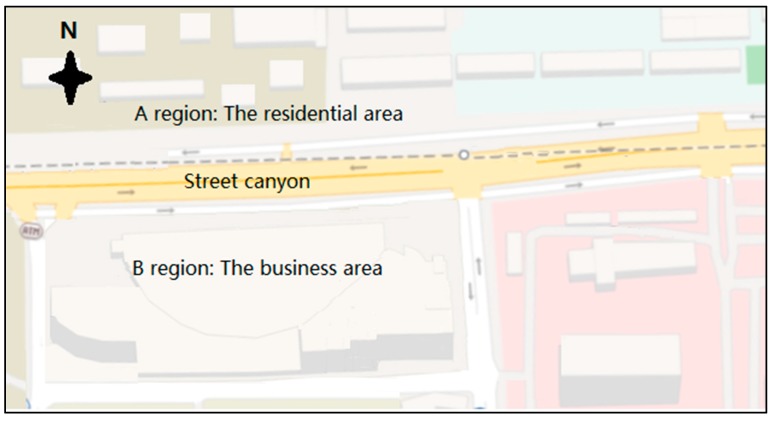
The street canyon in Luoyu Road, Hongshan District, Wuhan City (Baidu map).

The extension of No.2 metro line will add 13.35 km to the system which will be laid along the Luoyu Road of the Hongshan District of Wuhan City, as shown in Figure 1. The street canyon, connected to the roundabout in the west, along the railway line, comprises both residential and commercial buildings with different heights, which form into a typical asymmetric street canyon. This street canyon was selected as the case study due to the above characteristics. The north of the street canyon, identified as ‘A Region’, is a residential area with an average of 4 m gaps between buildings. The southern side, identified as ‘B Region’, is a commercial area with gaps between buildings ranging from 15 m to 20 m.

### 4.2. Geometric Model

#### 4.2.1. The Computational Domain

**Figure 2:**
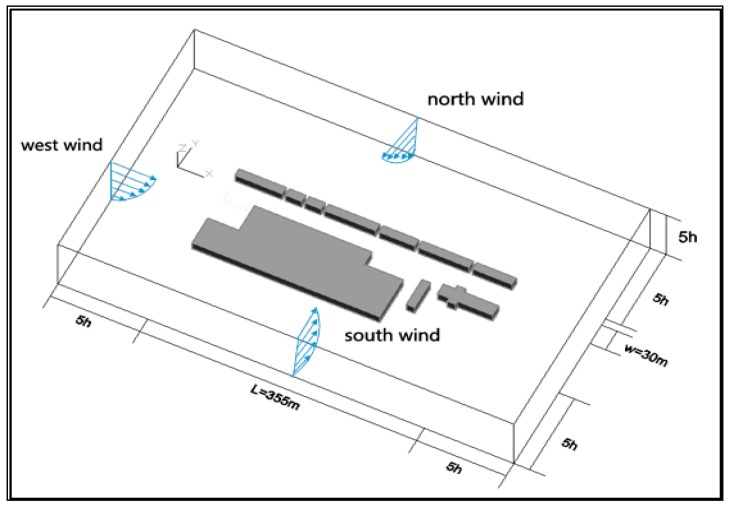
The computational domain.

The computational domain is represented in Figure 2. The selection of computational domain dimensions is undertaken according to existing guidelines for CFD simulation in the context of urban aerodynamics [39]. In the settings of the domain, h is the average height of buildings. The established street model extends the domain width in the x direction, with a consideration of long street canyons due to L/W > 10 [19]. A flow is identified at the inlet of the computational domain [34], and the simulations provide the flow with reference velocities as given in Section 4.3.1. In order to examine the impact of the height ratio, gaps between the buildings and wind directions on the street canyon, irregular shapes of adjacent buildings, i.e., arc corners etc., were simplified.

**Figure 3:**
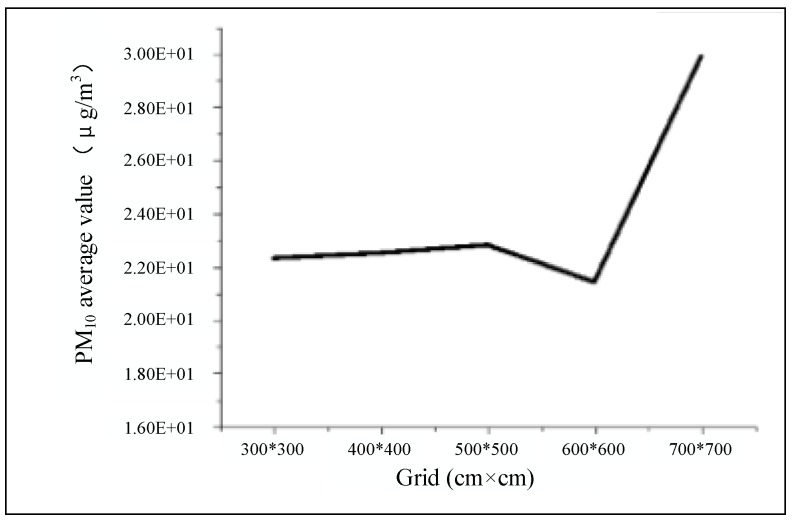
The relationship between grid size and average PM_10_ value.

The grid Ansys Workbench tool was used and a tetrahedral unstructured grid was applied for the spatial discretization of the computational domain. The computational domain is based on the design of the grid, and the number of cells and nodes remain same for the domains. The computational domain was 585 m long × 350 m wide × 145 m high and was discretized into 195 × 117 × 48 cells. The model boundaries were a distance 5 h from the horizontal domain, 5 h from the lateral domain, and 3 h from the vertical domain. A mesh sensitivity analysis, on the effect of the cell intervals, was conducted to verify the independence of the solution in order to confirm that the prediction result does not change significantly with different grid systems due to the COST guideline [47]. Figure 3 shows the relationship between the average PM_10_ value at 1.5 m high and the grid size. Average PM_10_ remains steady until the grid size of 500 cm × 500 cm, then drops slightly but shoots up at the grid size of 700 cm × 700 cm. The cell intervals near obstacles in the x, y, and z directions were △x = △y = △z = 0.33 h, respectively. The grid was fine enough to capture the physical phenomena in the model steadily. Therefore, the grid of 500 cm × 500 cm, with 2,375,100 cells in total, was selected after a mesh sensitivity analysis.

#### 4.2.2. The Base and Virtual Model

**Figure 4:**
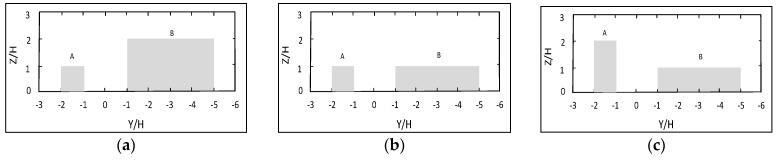
The street canyon and building models: (**a**) step-up model (H_A_/H_B_ = 0.5); (**b**) symmetrical model (H_A_/H_B_ = 1); (**c**) step-down model (H_A_/H_B_ = 2).

The symmetrical and asymmetrical canyons are classified by the ratio of the adjacent building heights (H_A_/H_B_) in Table 1 [19,48]. The width of the street canyon (W) is 30 m and the length (L) is 355 m. In the base model, the A Region is the residential area, the height of adjacent buildings is about 15 m (H_A_ = 15 m), and the building width is 15 m; therefore, H = 15 m was defined as the primary building height. While the B region is commercial, the height of the buildings is about 30 m (H_B_ = 30 m = 2H), and the building width is 60 m. Therefore, model 1, the base model, was considered as a step-up street canyon model (H_A_/H_B_ = 0.5). In order to investigate the distribution of particles in street canyons with different height ratios, model 2 (H_A_/H_B_ = 1) and model 3 (H_A_/H_B_ = 2) were established, as shown in Table 1. The street canyon models used in CFD simulation are shown in Figure 4. Therefore, the PM_10_ dispersion was investigated for the street canyon in the height ratios of 0.5, 1 and 2.

### 4.3. Parameter Definitions

#### 4.3.1. Wind Speed

The power law was adopted to address the value of wind speed. Based on local measurement campaigns, this assumed a reference velocity of 2.8 m s^−1^ at 10 m height. The wind profile was introduced as a user defined function (UDF) using the following formulation [49,50]:

(7) $$U_{Z}=U_{10}{\left(\frac{Z}{10}\right)}^{\alpha }$$

where: *U_Z_* (m s^−1^) is the wind velocity at height Z; *U*_10_ (m s^−1^) is the wind velocity at 10 m height; α is the roughness index decided by the topographical condition of buildings for the downtown area α = 0.23 [49,50,51].

#### 4.3.2. Emission-Source Rate

It is well recognized that heavy construction activities are largely responsible for emissions in urban street canyons [39,52]. Particulate matter with 10 μm in diameter was chosen as the main particle, since evidence shows that the more frequent dust events contribute much more to PM_10_ [53]. The construction of a rail transit system is a complicated process, and the mass of PM_10_ is mainly produced by soil excavation, earthmoving, the unloading of bulk materials, truck traffic and the emissions related to wind erosion and construction machinery in different phases [54]. In order to obtain the relatively sustained PM_10_ emission steadily, the process of soil excavation and earthmoving was chosen as the emission source, since research has suggested that these activities can easily account for up to 90% of the PM_10_ emissions for the single construction site [54].

The U.S. Environmental Protection Agency (USEPA) has developed the emission factor handbook (AP-42). This approach breaks down the factors of construction activities based on the generic operations, and the factor based on the site operation of soil evacuation and earthmoving is considered in this research [55,56]. It is modified by transferring the unit of emission factor from kg/t to g/m^3^ and two variables, i.e., earthwork density and height coefficient (HC) of the soil stevedoring were introduced to the original equation so that it can be used in the construction of both the road subgrade and rail transit, and the modified AP-42 method was employed in this study [57]:

(8) $$E_{D}=1.6\times k\times \rho \times \mathrm{HC}\times \frac{{\left(U/2.2\right)}^{1.3}}{{\left(M/2\right)}^{1.4}}=0.138\;g/m^{3}$$

where: $E_{D}$ is the particles emission factor of soil excavation and earthmoving operation (g m^−3^); k is the particle size coefficient; PM_10_ = 0.4; ρ is the earthwork density; 1.96 t m^−3^; HC is the height coefficient of soil stevedoring, HC = 2 m; U is the average measured wind speed during construction (1.8 m s^−1^); M is the surface material moisture content (13.2%); the average amount of soil excavation and earthmoving per unit time is 0.1 m^3^ s^−1^; and the intensity of the pollution source is 1.38 × 10^−2^ g s^−1^.

The emission source is located in a line along the north side of the street canyon, while the south side is used for regular city traffic, which is the main source of background concentration. Therefore, it is assumed that no other important sources of emissions except soil excavation and earthmoving in this process were identified in the research area; the only other values contributing to the PM_10_ concentration were background concentrations. Garcia, Cerdeira, Tavares, Coelho, Kumar and Carvalho [39] observed how changing building configurations of a real street canyon may affect the dispersion of PM_10_ by traffic. They found PM_10_ concentration at 1.5 m height is between 28 μg m^−3^ and 33 μg m^−3^ as background concentration. Also, there are 10 environmental monitoring stations set up by the Wuhan Central Meteorological Observatory and the air-quality data is reported hourly. The real-time PM_10_ data of the nearest monitoring station two kilometers away from this case is between 27 and 35 μg m^−3^. Therefore, the background concentration is considered 30 μg m^−3^ in this study. 

## 5. Validation of Numerical Simulation Results

**Figure 5:**
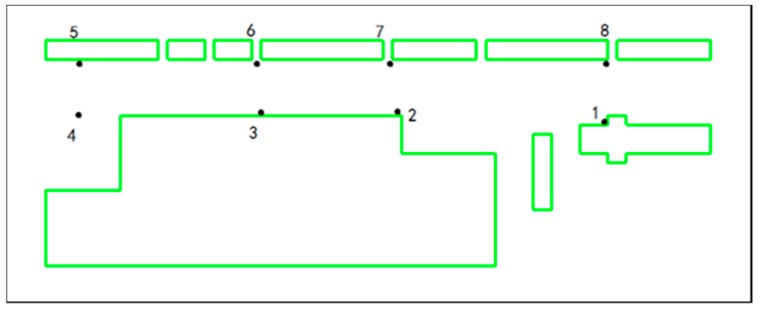
The sampling locations.

PM_10_ concentrations were measured during a field campaign, performed at Luoyu Road, Wuhan City, from 27 to 30 December 2016 between 2 pm and 4 pm (Beijing time). A NHFC79 dust monitor, calibrated at the place of measurement using the gravimetric method, was used to measure and record the concentrations of PM_10_. The equipment’s alarm set point is from 0 to 999 μg m^−3^ and displays the statistics of maximum, minimum and average readings and elapsed time. The dust monitor was set periodically at eight different sampling points at 1.5 m height (Z = 0.1H) above ground level along the canyon length. This is a typical human breathing height for exposure [39]. The measurement at each point lasts for about 5–8 min, which is exclusive of preparation. Figure 5 shows the sampling locations.

A summary of max, min and average measured PM_10_ concentrations and numerical simulations is provided in Table 2. Beyond the background concentration, only PM_10_ emissions from construction were considered for numerical simulation. Meteorological data was provided by Wuhan Central Meteorological Observatory. The average ambient temperature was 9 °C, average wind speed was 1.8 m s^−1^ at 1.5 m height, and relative humidity during the measurement campaigns was 54.6 RH in the field campaign period. A neutral inflow condition is used in this research, and the skimming flow, e.g., 1.8–6 m s^−1^, tends to be less disturbed, which is considered as a relatively stable atmospheric.

Table 2 shows the values of modelled PM_10_ concentrations at 1.5 m height for model 1. Measured PM_10_ concentrations are reported at all eight sampling points located in the street canyon (see Figure 5). The highest PM_10_ concentrations in numerical simulation were found at point 1 with a value of 51 μg m^−3^ with a condition of parallel wind. This point is located at the east end of the street, near the large gaps in the southern side. This point also shows the highest value in measurements. A linear regression analysis with three statistical parameters has been calculated, and a good correlation (R = 0.97) in addition with RRMSE = 0.06 and bias = 0.03 are obtained, indicating these values are reasonably close to each other. The numerical simulation results show a slight under measurement at points 4 and 5 with differences of 10.7% and 12.5%, respectively. The street canyon connects to the roundabout in west, and car traffic would make the background concentration higher in the west. This is arguably the reason for the larger differences at points 4 and 5.

## 6. Results

### 6.1. Influence of Height Ratio

#### 6.1.1. Horizontal Plane for Wind from West

**Figure 6:**
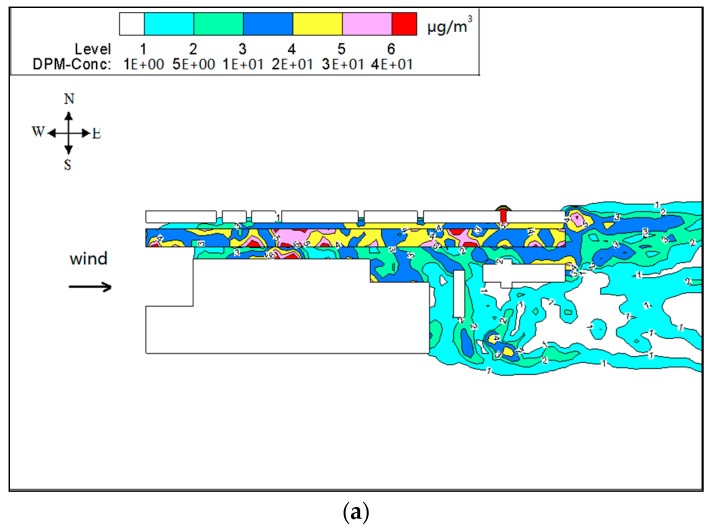

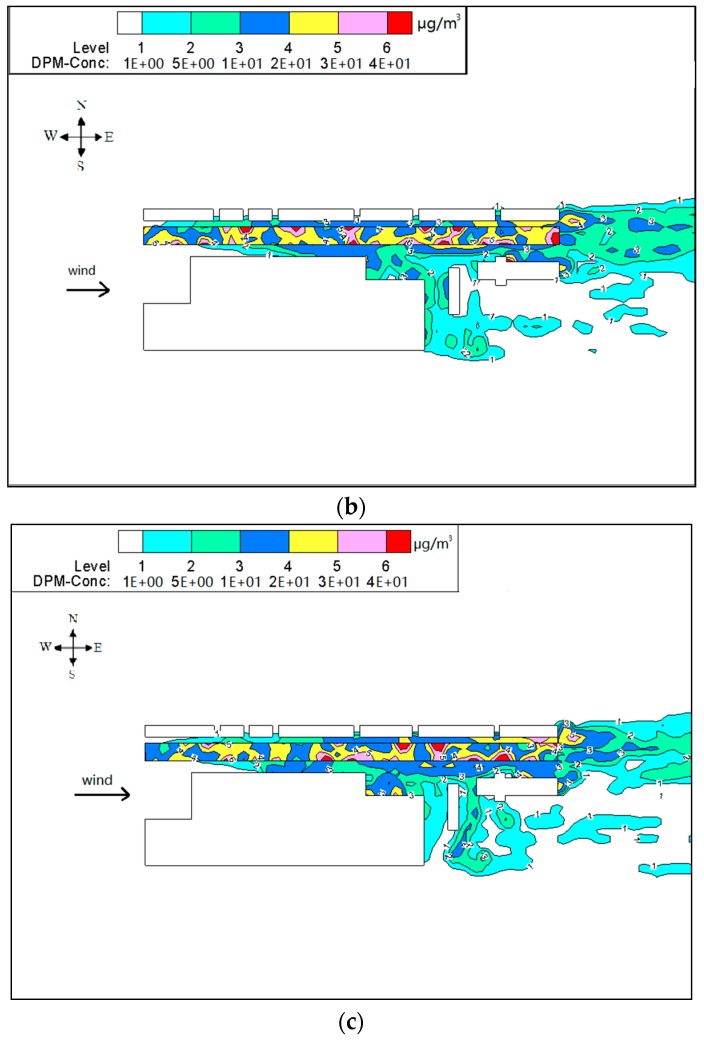
Contours of PM_10_ in models 1 (**a**), 2 (**b**) and 3 (**c**) for wind from west (Z = 0.1H).

**Figure 7:**
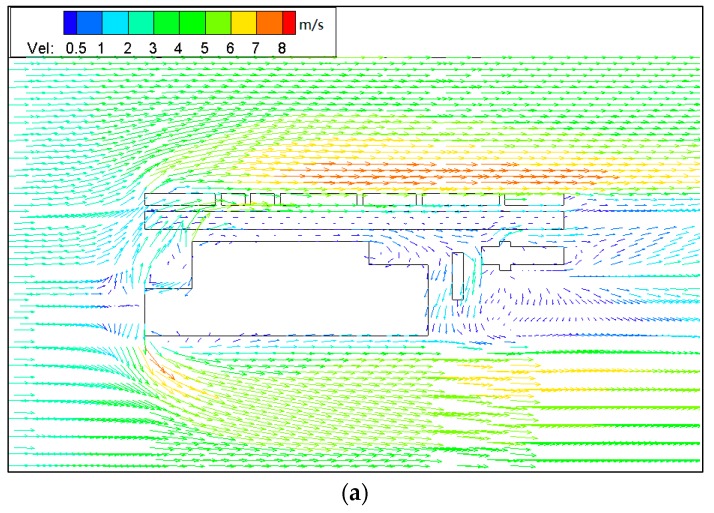

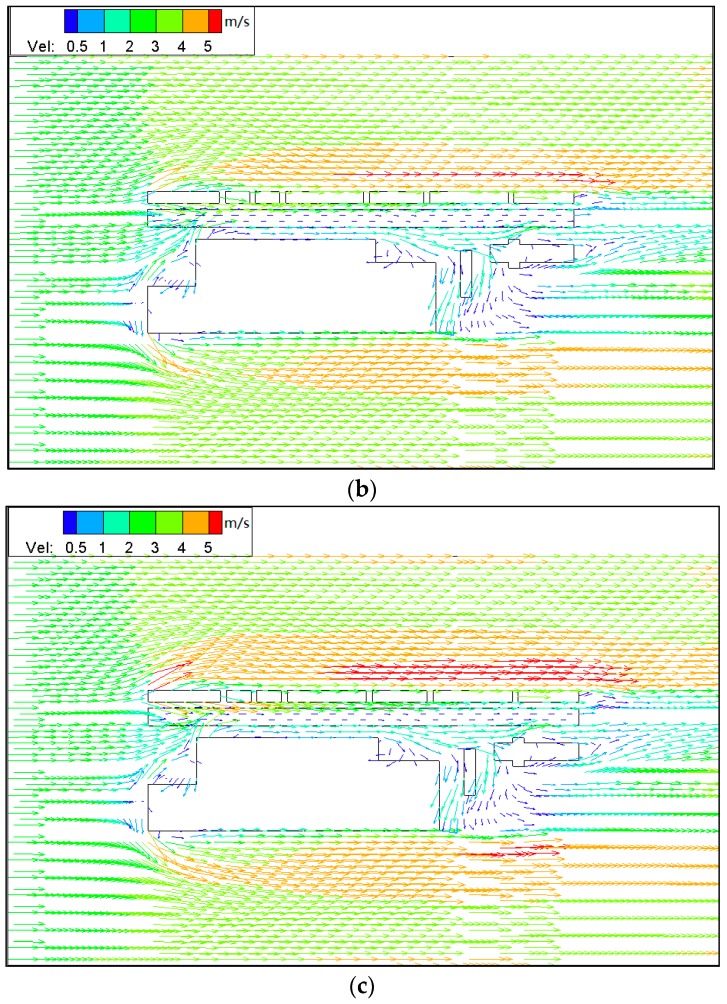
Streamlines of wind speed in models 1 (**a**), 2 (**b**) and 3 (**c**) for wind from west (Z = 0.1H).

Figure 6 and Figure 7 show PM_10_ contour plots and streamlines of wind speed at Z = 0.1H height due to emissions from construction activities for the step-up street canyon (model 1), symmetrical street canyon (model 2) and step-down street canyon (model 3), respectively. The result of concentrations for a horizontal plane shows that the configuration with different height ratio presents similar characteristics of concentrations. The concentrations of PM_10_ appear in the middle and the end of the street canyon (see Figure 6). There is a wider range of pollutant dispersion in the gap of the B Region in the step-up model (see Figure 6a). Moreover, lower concentration appears in south part of the street in all three models. It shows that a west wind was divided into several parts when flowing through the beginning of the street canyon (see Figure 7), and due to the roughness effect, the inflow decelerates along the canyon.

#### 6.1.2. Vertical Plane for Wind from West

**Figure 8:**
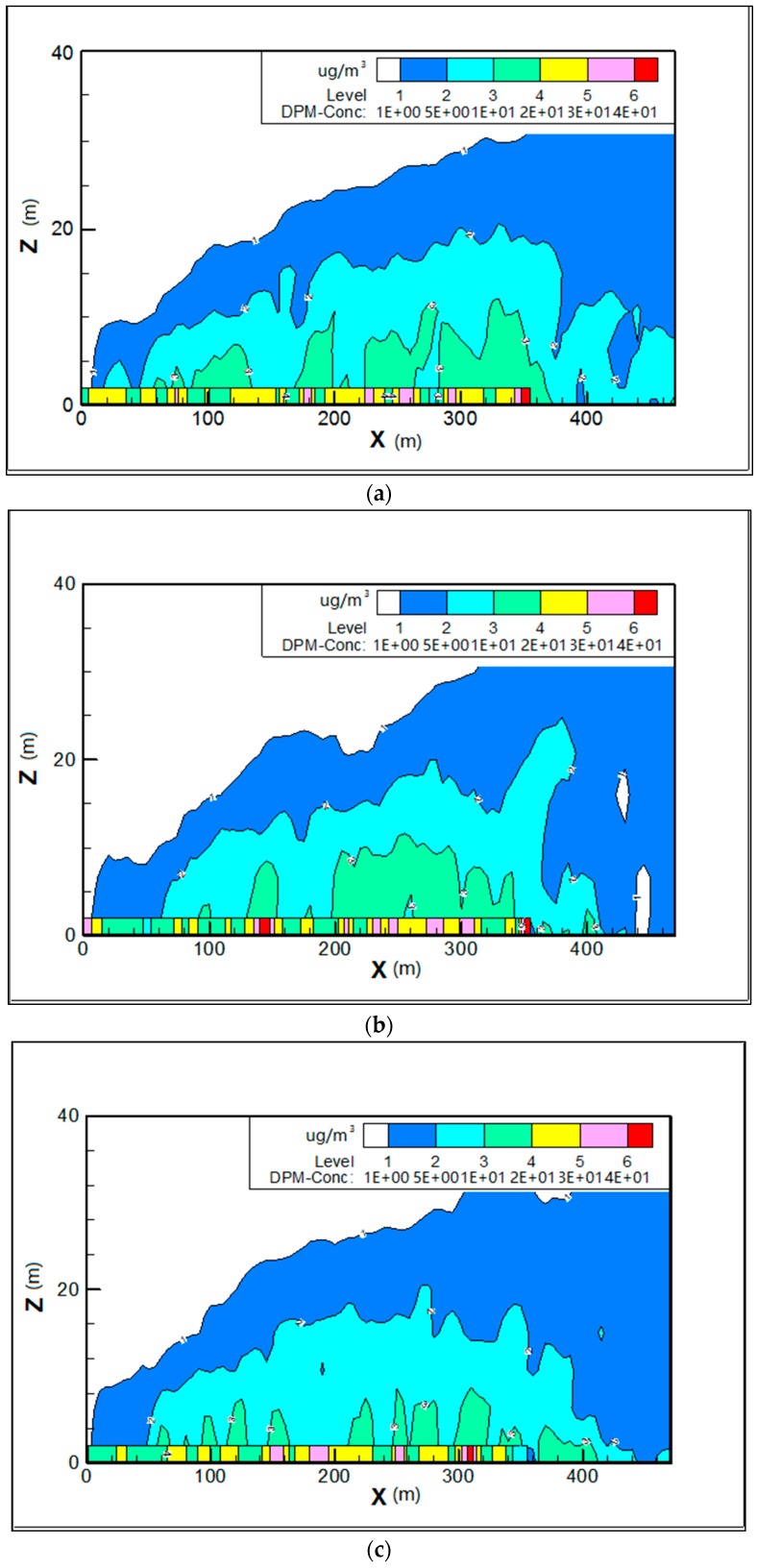
Contours of PM_10_ in the vertical center plane (Y = 0) for wind from west in models 1 (**a**), 2 (**b**) and 3 (**c**).

**Figure 9:**
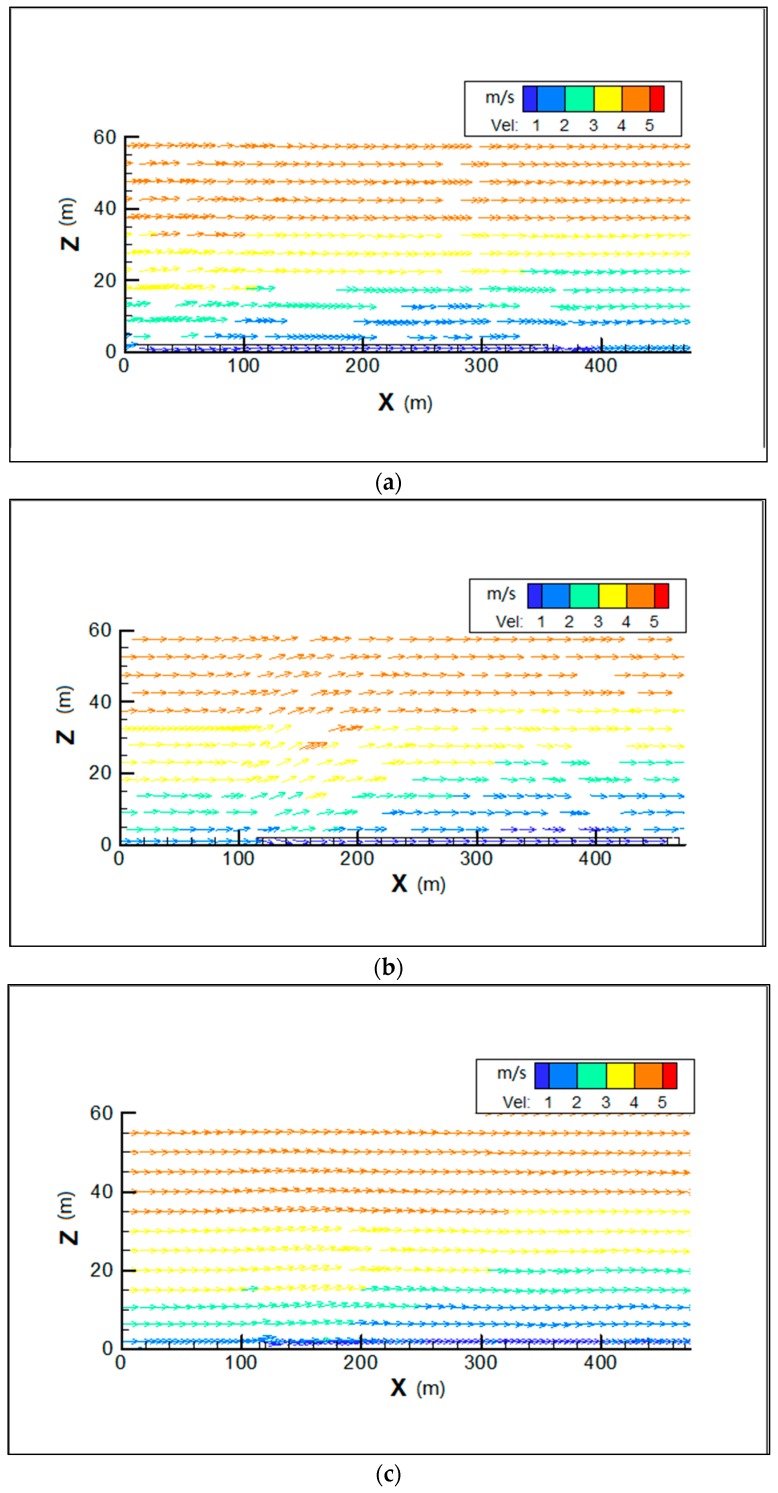
Streamlines of wind speed in contours of PM_10_ in the vertical center plane (Y = 0) for wind from west in models 1 (**a**), 2 (**b**) and 3 (**c**).

In order to investigate the concentration and dispersion of PM_10_ at vertical heights, the vertical plane in the center of the street (Y = 0) was set up. Figure 8 shows the differences on vertical dispersion. In all the three models, the influence of PM_10_ is below 20 m on average on vertical dispersion. Concentration profiles were similar in the step-up (see Figure 8a) and step-down street canyon models (see Figure 8c), and while either in concentration or the vertical direction it indicates a slight strong PM_10_ dispersion in the symmetrical model (see Figure 8b). Figure 9 showed that pollutants were brought to the air when the flow meets the street canyon and part of the flow moves around it. It also shows that the direction of flow above the roof is in accordance with that inside the street canyon. The air flows along the street in the step-up (Figure 9a) and step-down street canyon models (Figure 9c) are less disturbed, as the adjacent building has the smaller height (H = 15 m) in these two cases, which leads to the lower concentration of PM_10_ in the middle and end of the street in the vertical direction.

#### 6.1.3. Lateral Plane for Wind from West

**Figure 10:**
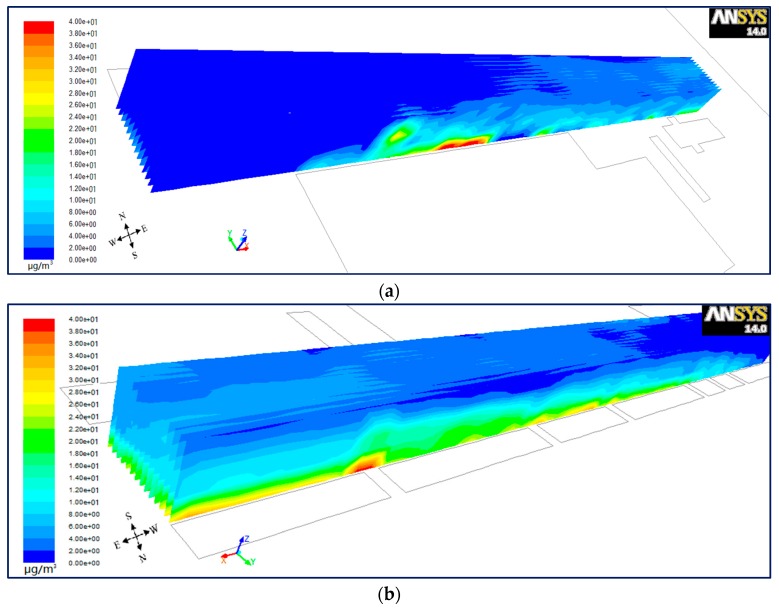
Bird’s eye views on PM_10_ distributions from the south-west (**a**) and north-east (**b**) for wind from the west in model 1.

**Figure 11:**
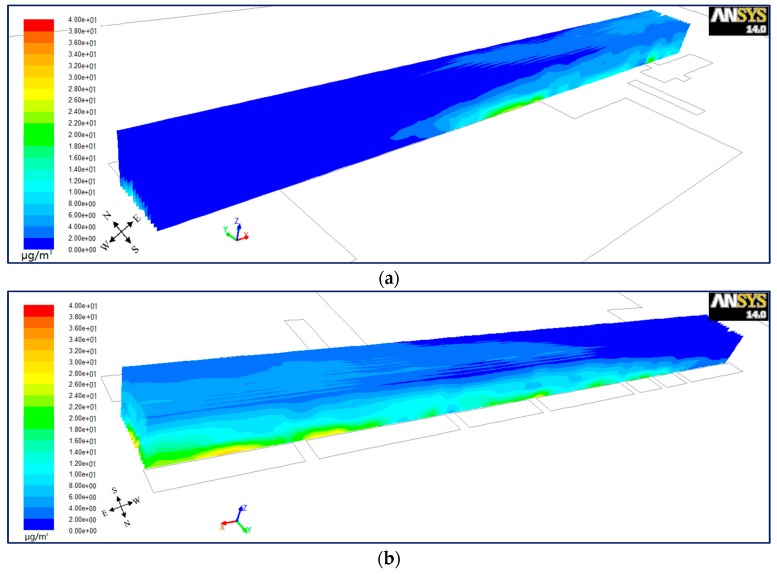
Bird’s eye views on PM_10_ distributions from the south-west (**a**) and north-east (**b**) for wind from the west in model 2.

**Figure 12:**
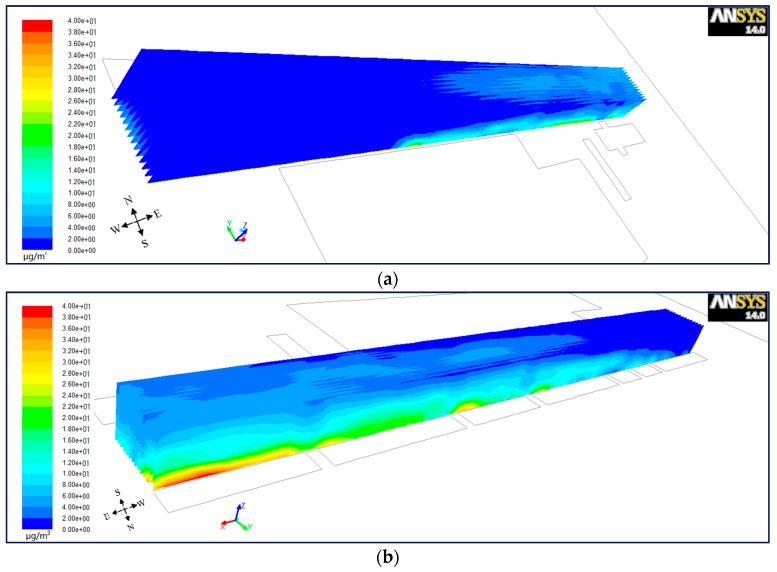
Bird’s eye views on PM_10_ distributions from the south-west (**a**) and north-east (**b**) for wind from the west in model 3.

In order to investigate the differences of PM_10_ dispersion from north to south inside the street canyon, the lateral planes were set up every 2 m (*Y* = 13 − 2*n*, *n* = 0, 1, 2……13). Figure 10, Figure 11 and Figure 12 present a bird’s eye views of PM_10_ distributions from the south-west (a) and north-east (b) for wind from the west in the step-up (see Figure 10), symmetrical (see Figure 11) and step-down (see Figure 12) models. It shows that for vertical as well as horizontal directions, there is a higher concentration of PM_10_ in the north than the south of the street in all three models. However, for parallel wind, the difference of concentration between north and south is not prominent in symmetrical street canyons compared to asymmetrical ones. Figure 7 in Section 6.1.1 shows the cavity in the upwind direction affects the inflow when it travels through the street canyon in parallel flow, which gains speed in the north compared to the south, and causes higher concentration. But the difference of wind speed in the symmetrical street canyon model (see Figure 7b) is smaller than that of the asymmetrical models (see Figure 7a,c). This illustrates that the effects on the imbalance of wind speed on the north and south sides by cavity in the upwind direction is stronger in the asymmetrical model than that in the symmetrical model, and the cavity in the south will accelerate the inflow in the opposite direction.

### 6.2. Influence of Wind Direction on Distributions of PM_10_

Previous studies using numerical analysis have suggested that it is a complex process for the transition of flow structure from parallel flow to perpendicular flow in regular (H/W = 1) and short canyons (L/W = 1) [19,41,42]. Therefore, the next section reports the results of PM_10_ dispersion in the perpendicular flow from the north and south in long street canyons (L/W = 12.2).

#### 6.2.1. Distribution of PM_10_ and Streamlines of Wind Speed for Different Wind Directions

##### North Wind Direction

**Figure 13:**
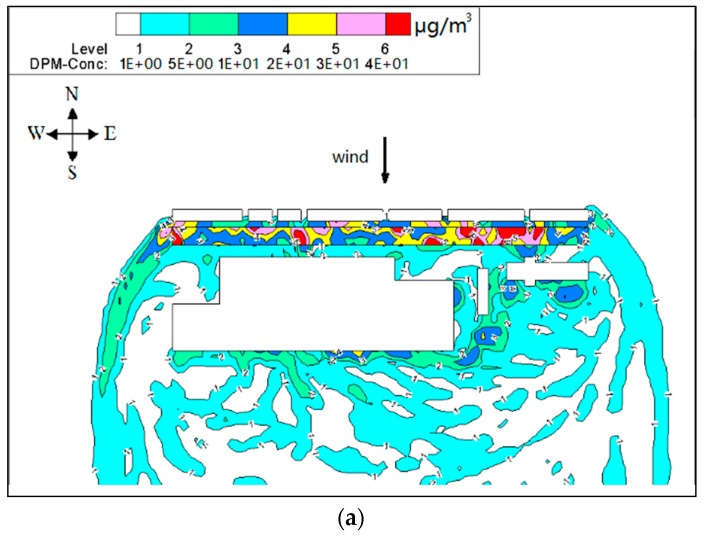

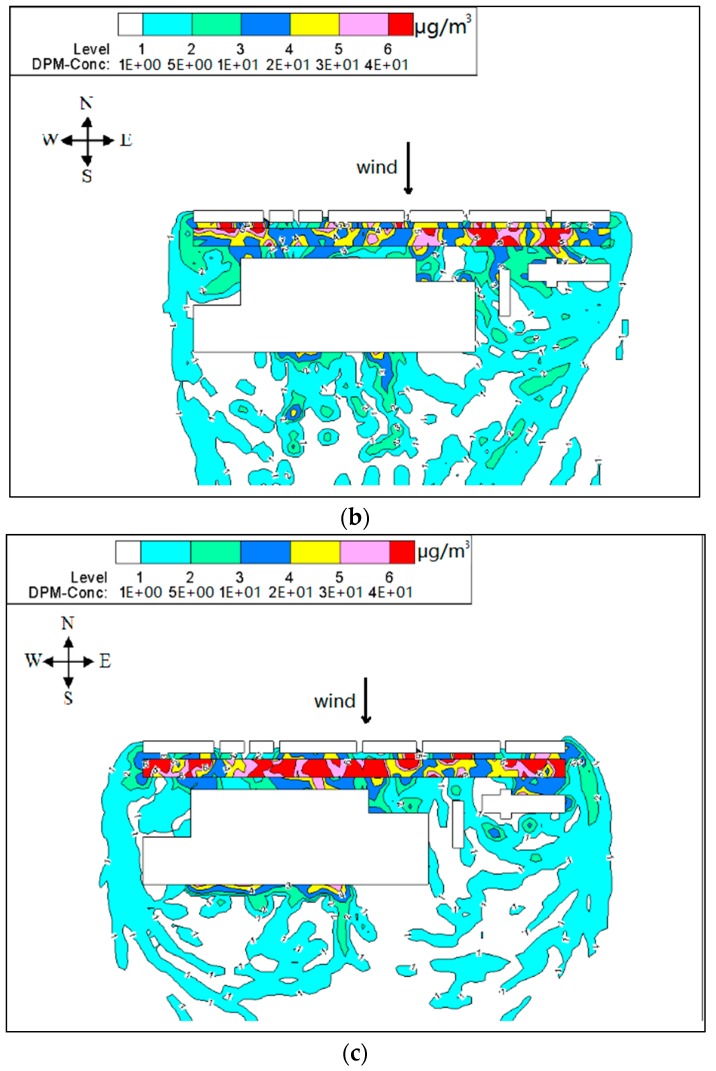
Contours of PM_10_ in models 1 (**a**), 2 (**b**) and 3 (**c**) for wind from the north (Z = 0.1H).

It is possible to observe in Figure 13 that there is higher concentration of PM_10_ in all the models for wind from the north. However, compared with the step-down model (see Figure 13c), the step-up street canyon (see Figure 13a) helps the PM_10_ disperse from inside to outside, suggesting higher concentration for higher height ratio in the north wind condition. Furthermore, PM_10_ concentrations are likely to occur in the leeward side of upwind buildings. Using wind-tunnel measurement, some studies have shown that the pollutant concentration in a regular street canyon declined gradually from the bottom, whereas the pollutant level at the leeward wall is higher than that at the windward wall [57,58].

**Figure 14:**
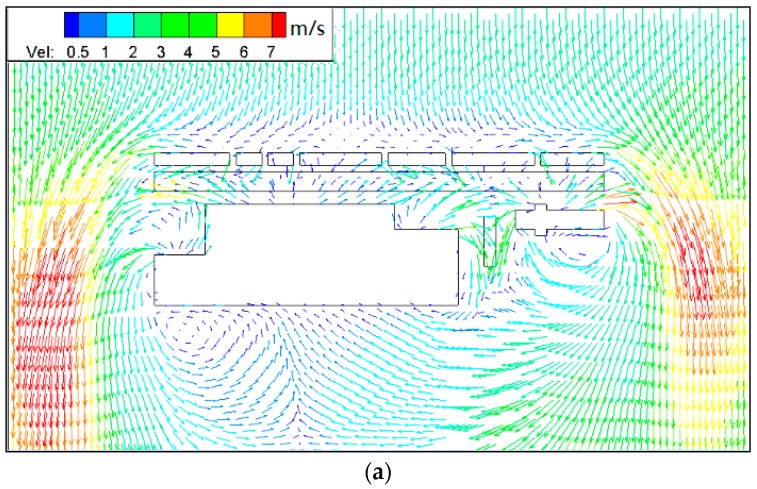

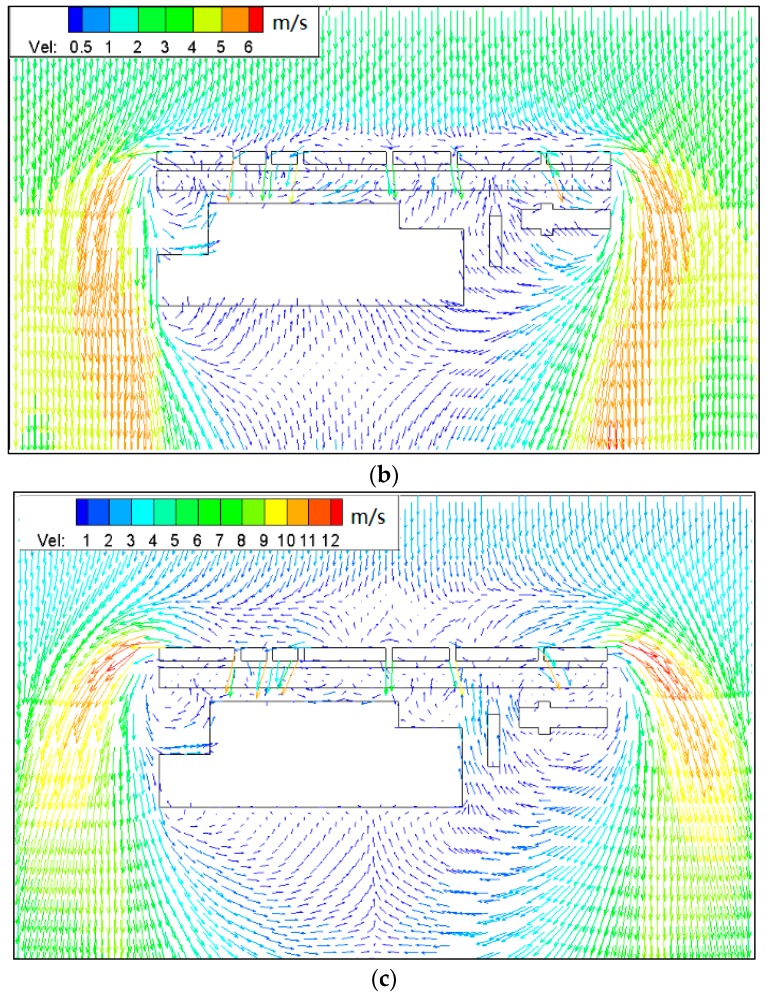
Streamlines of wind speed in models 1 (**a**), 2 (**b**) and 3 (**c**) for wind from the north (Z = 0.1H).

Figure 14 shows the streamlines of wind speed in street canyon models. It indicates that the stream from the north impacted against the buildings in region A and diverged into two substreams passing by the canyon perpendicularly, forming double-eddy circulations outside the beginning and end of the canyons. On the one hand, this will bring PM_10_ back to the canyon through the gap in the south. On the other hand, PM_10_ will accumulate at the leeward side of both northern and southern buildings and the concentration will become higher with the increase in height ratio.

##### South Wind Direction

**Figure 15:**
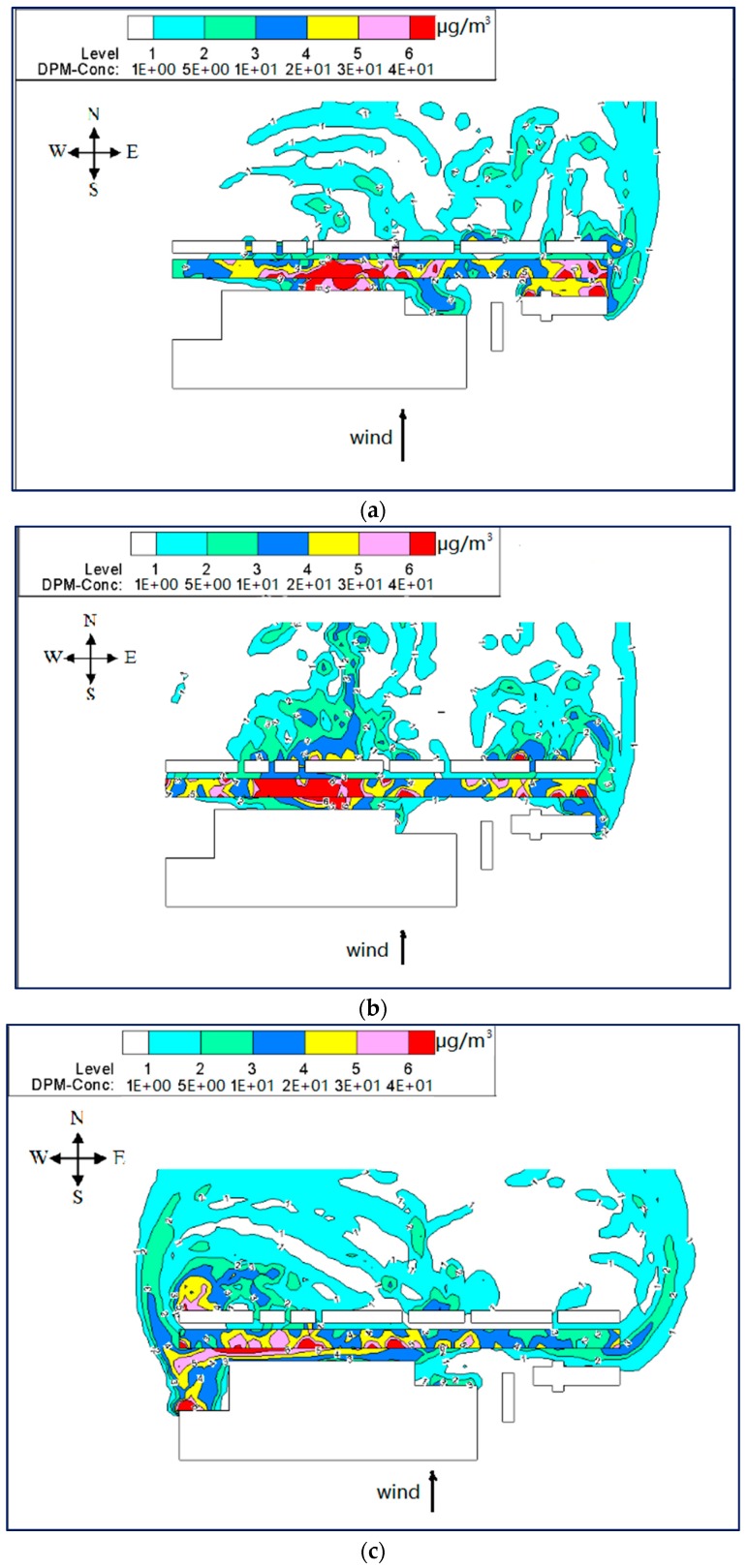
Contours of PM_10_ in models 1 (**a**), 2 (**b**) and 3 (**c**) for wind from the south (Z = 0.1H).

Figure 15a,b indicate that PM_10_ concentrations were quite similar in the middle of the street canyons, while Figure 15c shows that the high concentration in the beginning and middle parts. Moreover, higher PM_10_ concentration was also observed in the cavity at the beginning of the street canyon in model 3 (see Figure 15c). There are comparatively higher concentrations in the step-up (model 1) and symmetrical street canyons (model 2), while the pollutant accumulates in the leeward of downwind buildings in the step-down street canyon (model 3).

**Figure 16:**
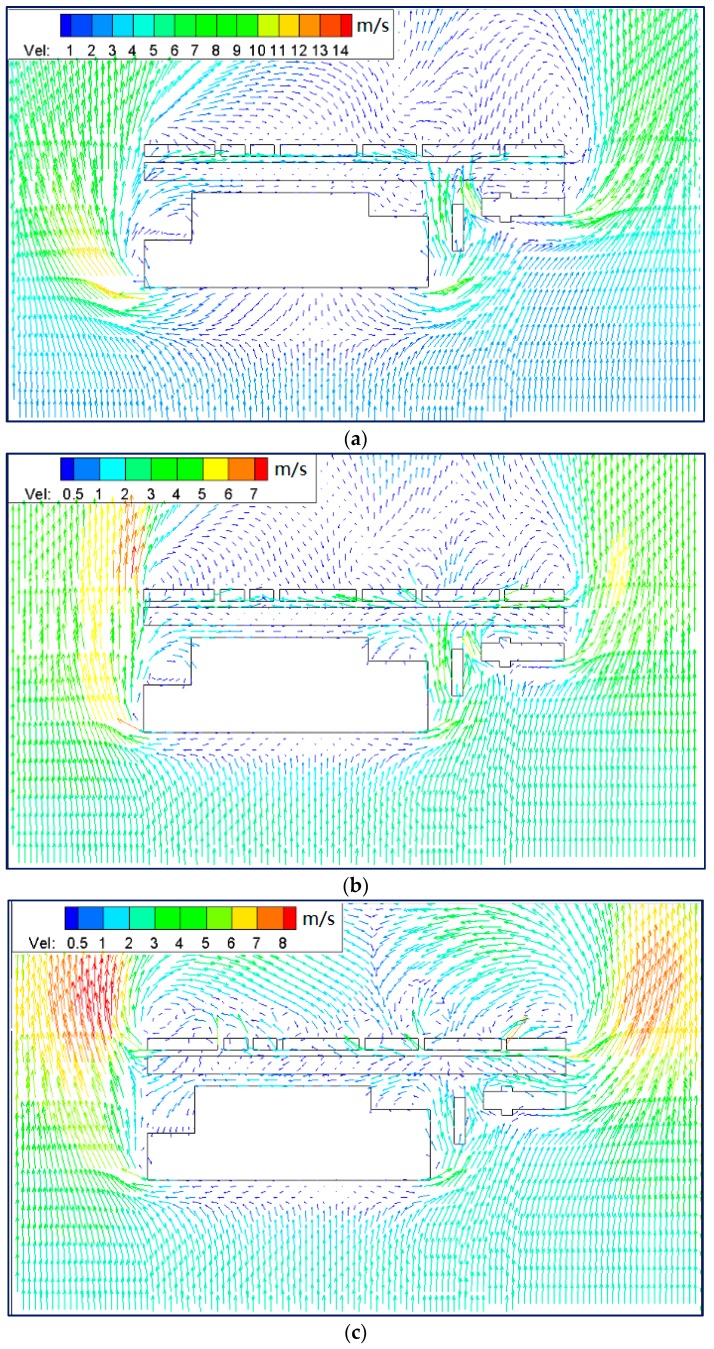
Streamlines of wind speed in models 1 (**a**), 2 (**b**) and 3 (**c**) for wind from the south (Z = 0.1H).

Figure 16 presents the double-eddy circulations outside the beginning and end of the canyons. Furthermore, it indicates two substreams converge together in the middle of the street canyon because of the higher adjacent buildings (see Figure 16c), which led to a stronger accumulation of PM_10_ leeward of northern buildings. Meanwhile, due to the large gaps in southern buildings, there also exist double-eddy circulations in the street canyon. The effects of circulation on the three models are not similar. For the condition of wind from the south, the substream went into the canyon from west to east in the step-up street canyon model (see Figure 16a); from both sides in the symmetrical street canyon model (see Figure 16b); and from the gaps in the south that went out of the canyon from the west side (see Figure 16c). Therefore, a higher concentration of PM_10_ is found in the middle of the canyon in models 1 and 2, and the cavity of the west side in model 3. 

It is concluded that for the condition of parallel wind, the double-eddy circulations are usually observed outside the canyon. This finding is similar to those reported by Yazid, Sidik, Salim and Saqr [19]. Their review of existing studies concluded double-eddy circulations often located along the corner of the street canyon end for perpendicular wind. However, the current study found that there are also the double-eddy circulations existing in the canyon, if there are large gaps in the upwind buildings. Furthermore, the height ratio shows the different pollutant dispersion characteristics, while there are higher concentrations in the street canyon and on the leeward of backwind buildings, when the upwind building is taller.

#### 6.2.2. Mean Values of PM_10_ for Different Wind Directions

In order to investigate the characteristics of pollutant dispersion in all models for different wind directions, mean values of PM_10_ in the vertical plane (Z = 0.1H) were measured, as shown in Table 3. Mean value is the area-weighted average PM_10_ computed by dividing the summation of PM_10_ by the total area of the surface, covering 0 ≤ x ≤ 355 m and −15 m ≤ y ≤ 15 m. The concentration of PM_10_ was found to be similar for the parallel flow while quite different for the perpendicular flow. The PM_10_ concentration is highest in the step-down street canyon (model 3) for the north wind conditions. By contrast, the most polluted situation occurs in the step-up street canyon (model 1) for the south wind conditions. This indicates that for perpendicular flow, the taller the upwind building the higher the pollutant concentration, which is in accordance with the results in Section 6.1. Moreover, the worst situation is observed for the north wind in the step-down street canyon (model 3).

#### 6.2.3. Mean Values of PM_10_ in Horizontal, Vertical and Lateral Planes for Different Wind Directions

In order to obtain the distribution of PM_10_, mean values of PM_10_ were calculated along specific distances of the X, Y and Z axis.

**Figure 17:**
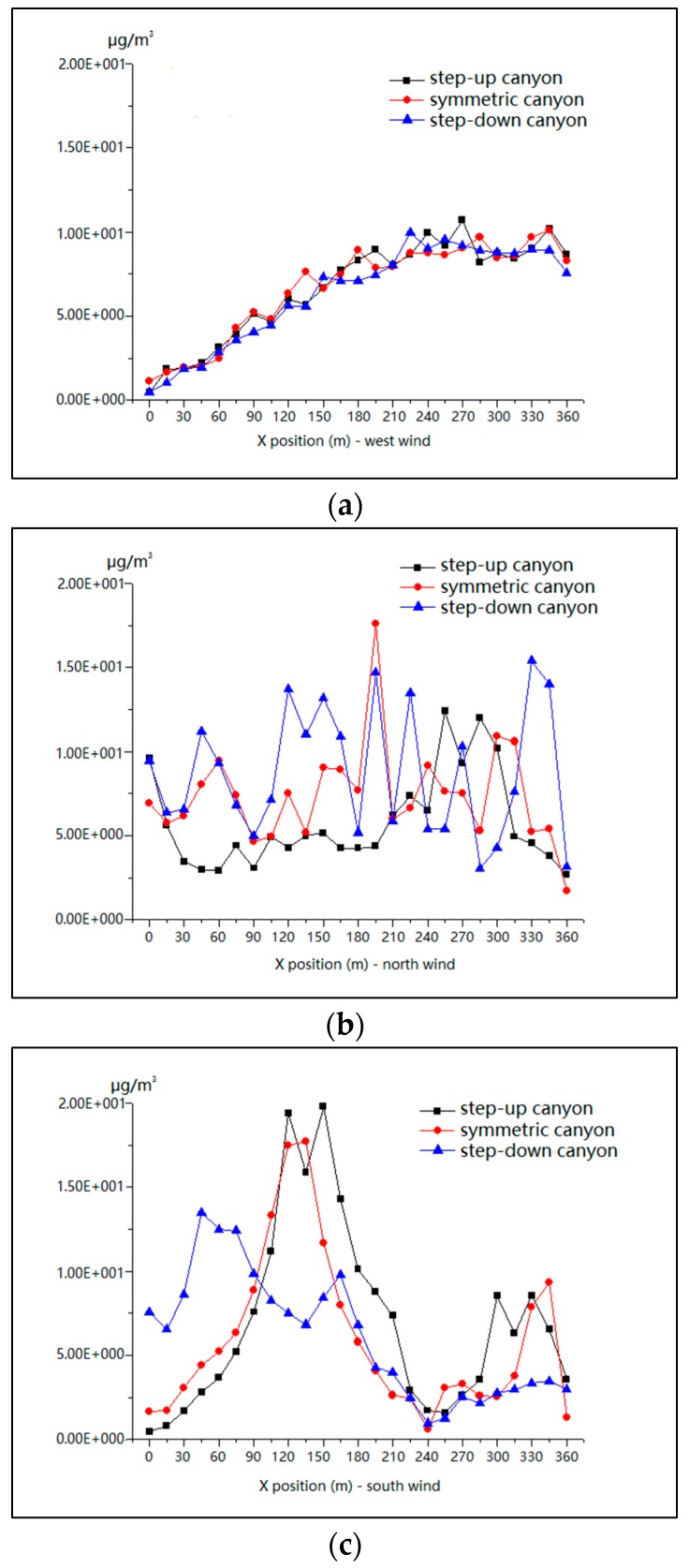
Mean values of PM_10_ in model 1, model 2 and model 3 for wind from the west (**a**), north (**b**) and south (**c**) along the X-axis.

As demonstrated in Figure 17a for the condition of the west wind, mean values are close to each other in all these three types of street canyon, and they are increasing along the X axis. This shows that there are no significant differences among the street canyons for the parallel flow condition. However, there are no special trends for the north wind for all models (see Figure 17b). This is affected by the gaps in northern buildings and, as a result, the mean values fluctuated considerably. For the condition of the south wind, the analysis reveals two peaks in the middle of the step-up and symmetrical street canyons (see Figure 17c), and a trough corresponding to the gap in southern buildings in all the street canyon models. This indicates a strong impact of height ratio on PM_10_ concentrations in the middle part of the step-up and symmetrical street canyons for wind from the south.

**Figure 18:**
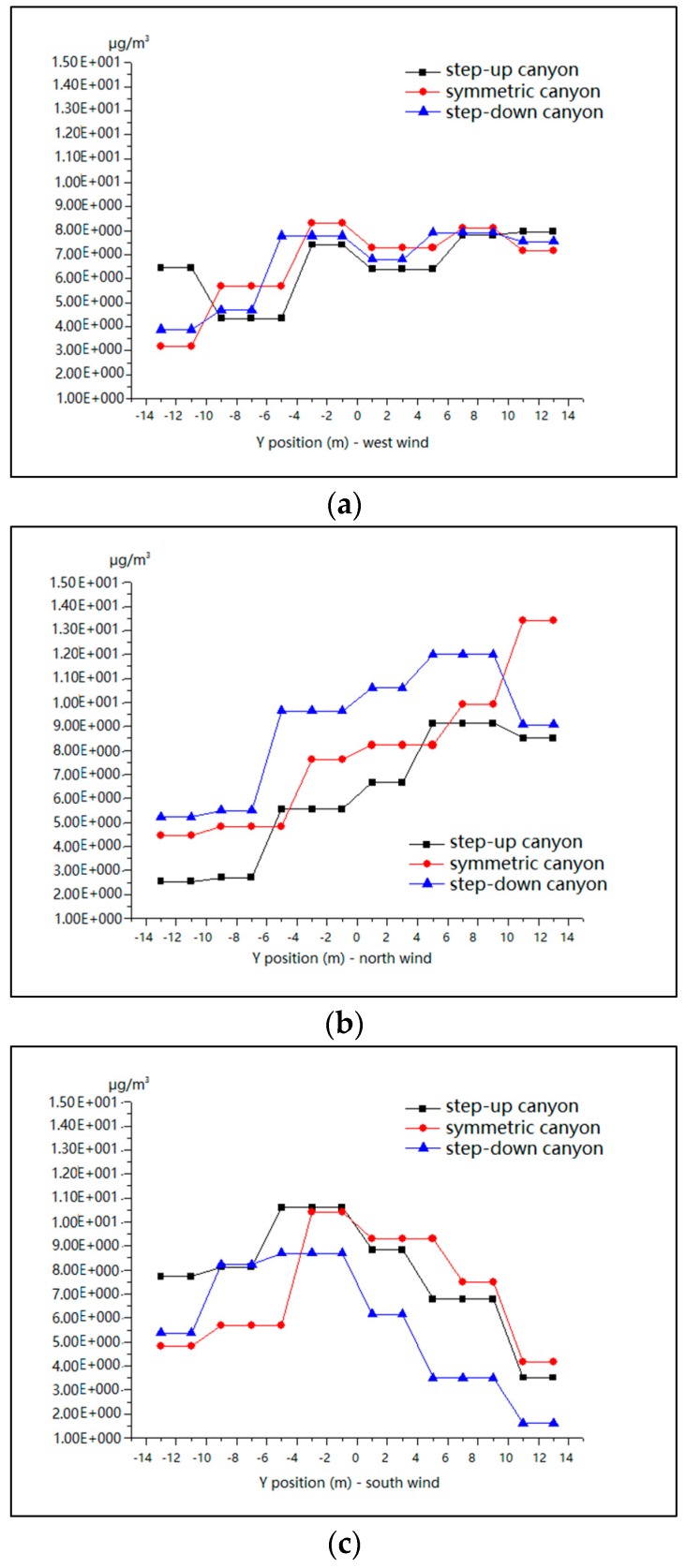
Mean values of PM_10_ in model 1, model 2 and model 3 for wind from the west (**a**), north (**b**) and south (**c**) along the Y axis.

Figure 18 indicates a similar trend of the distribution of PM_10_ from south to north in the street canyons along the Y axis in all models. The concentrations become gradually higher from south to north for wind from the west (see Figure 18a), a sharp increase for the north wind (see Figure 18b), and an inverted U-shaped curve for the south wind reaching the peak at the center (see Figure 18c).

**Figure 19:**
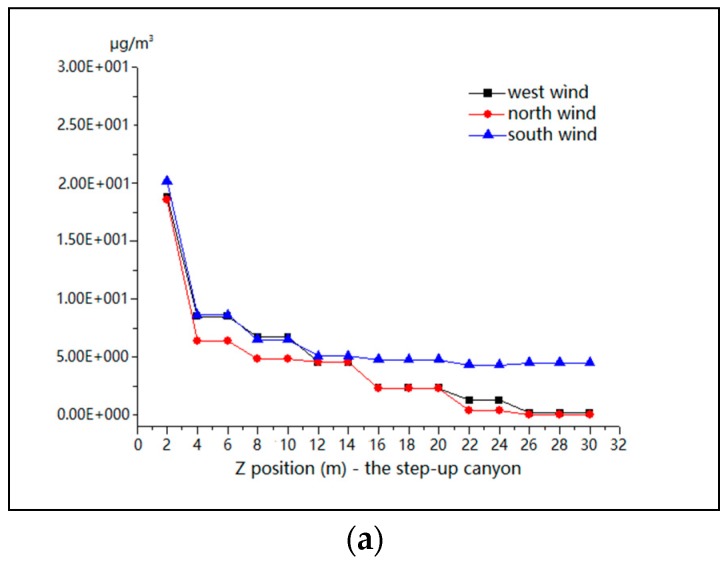

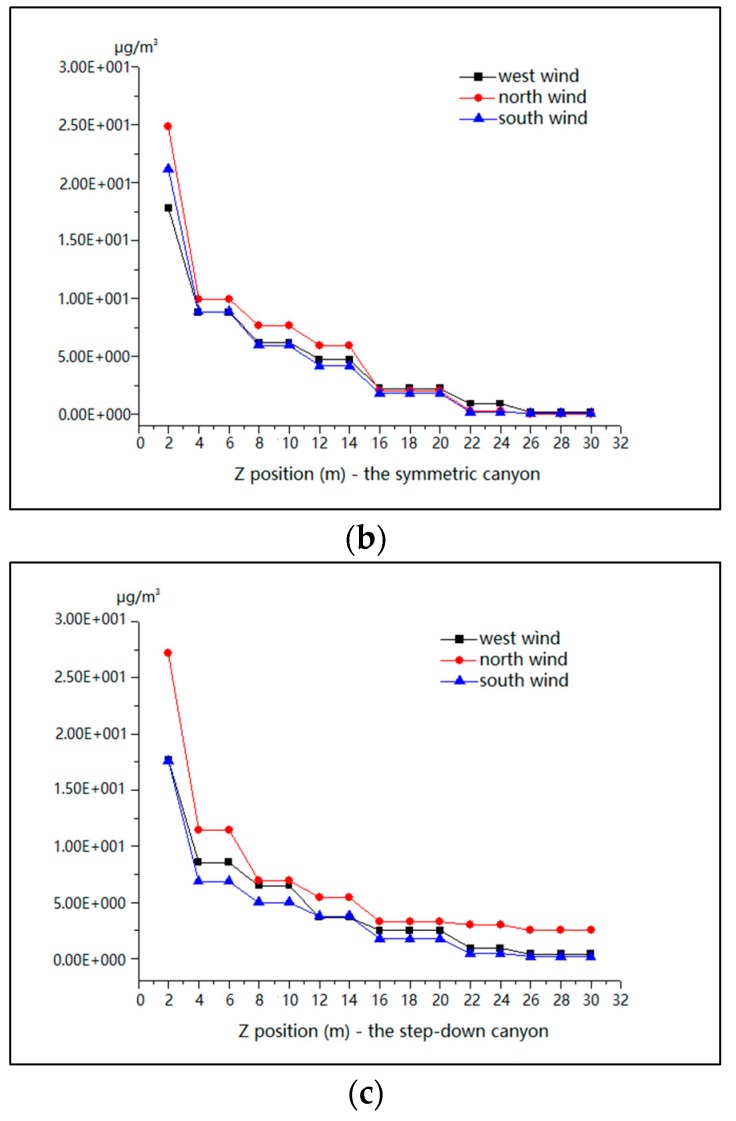
Mean values of PM_10_ for wind from the west, north and south in model 1 (**a**), model 2 (**b**) and model 3 (**c**) along the Z axis.

Figure 19 illustrates the distributions of PM_10_ in vertical directions along the Z axis for different wind directions for all three street-canyon models. In general, the concentrations have the same trend as the simulated data with values decreasing sharply in the first 4 m, falling gradually with height, and remaining stable above 20 m high. Similar findings were reported in past studies where the pollutant dispersion decreases exponentially [20]. Therefore, mitigation measures should be taken in all types of street canyons for all wind directions below 20 m, specifically below 4 m.

## 7. Discussion

Results of this study showed that pollutants concentrate at the middle and end of a street canyon if the direction of the wind is similar to that of the street canyon. This is in line with those studies using wind-tunnel experiments [59]. In a previous study, Assimakopoulos, ApSimon and Moussiopoulos [38] reported the wind-field and pollution-dispersion pattern is a major vortex which covers the top end of the street canyon. 

When the flow is perpendicular to the street axis, results showed that the pollutant concentration was high at the leeward wall. This is similar to the work on the RANS turbulence model conducted by Baik and Kim [60]. The change in wind direction causes the change in flow structure and the dispersion of pollutants along the street canyon [60].

The influence of PM_10_ in the vertical plane is mainly below 20 m height in both parallel and perpendicular flows. Within the range from 1.5 to 4 m, the concentration of PM_10_ declined significantly along the height, and changed slightly in the range of 4–20 m, whereas it remained stable above 20 m. It has been reported in previous studies on particles of nucleation and Aitken mode that the peak concentrations of the nucleation mode (diameter < 0.02 μm) and the Aitken mode (0.02 μm < diameter < 0.1 μm) declined significantly along the height and remained stable in the range of 8–20 m [23]. Further study on the number of particles at different heights in the street canyon suggested that the concentration dropped significantly when the height increases during the day; however such a gap is much smaller in the perpendicular flow [61]. As to the particle dispersion in the vertical plane, some experimental investigations reported that pre-existing conditions play a crucial role in the density of particles [62]. For instance, the particle grows by aerosol dynamic processes when transporting beyond the street canyon due to the pre-existing particles at the rooftop level [63].

This study also illustrated that the average PM_10_ concentrations rise about 1.5 times at 1.5 m height when the wind direction changes from parallel to perpendicular flow due to poor dispersion. This is in line with the findings of Longley, Gallagher, Dorsey, Flynn, Allan, Alfarra and Inglis [64]. Their study showed that the average value increased about three times when the wind direction changed. 

Results also showed that the concentrations of total particles varied according to wind direction and height ratio. If a downwind building is higher than an upwind building, when the wind blew perpendicular to the canyon, the pollutants released inside the street canyon are diluted very effectively. This is in line with findings of the numerical tests reported by Huang, Akutsu, Arai and Tamura [65]. 

As to the modeling based on the real street canyon, the irregular street and buildings as well as gaps are significant factors that affect the flow structure. In this study, different gaps existed in adjacent buildings on both sides of the street canyon. This will help dilute pollutants with an average of 4 m gaps in upwind buildings. This finding is in accordance with those reported by Garcia, Cerdeira, Tavares, Coelho, Kumar and Carvalho [39] who investigated the influence of virtual changes in building configurations of a real street canyon on PM_10_ dispersion. They suggested that gaps between buildings improved PM_10_ concentrations during cross-canyon winds. However, no significant improvements were noted with 6 m-wide gaps compared to 4 m for the same wind direction. Furthermore, double-eddy circulations are observed inside the canyon with an average 15–20 m gaps in upwind buildings in this study. This illustrates that if the gap is large enough, it will divide the long canyon into several short ones, which forms the additional double-eddy circulations and changes the flow structure and the position of the concentration.

The CFD model in this study is based on a true street canyon, and the results may be affected by some factors, such as the building width configuration, regularity of buildings, streets, gaps and etc. This study only took perpendicular and parallel flow into consideration. This is because most CFD simulations have already covered the best and worst wind direction for pollutant dispersion inside the street canyon [48]. Despite this, it is still worth examining the result in terms of oblique wind directions and the change of wind speed. In addition, only the height ratio configuration is examined in this study. It is well known that the aspect ratio is considered as the main configuration of a street canyon, which is fully developed in the existing literature. Therefore, this study focuses on the influence of height ratio to particle dispersion in a street canyon. Future research is warranted to validate these findings by considering the impacts of both aspect ratio and height ratio.

## 8. Conclusions

Heavy construction activities have significant effects on particle emissions. Street canyons present a good opportunity to understand the local atmospheric flow in the built environment. This study focuses on PM_10_ mass concentration and dispersion due to construction activities by examining three height ratios (0.5, 1 and 2). These height ratios are crucial for the accurate CFD simulation of perpendicular and parallel flow inside a street canyon [34].

This study revealed the different influence of height ratios in the geometry configuration and wind direction on PM_10_ dispersion. A perpendicular flow would lead to the accumulation of PM_10_ in the end of the street canyons under the height ratios of 0.5, 1 and 2. Meanwhile, for parallel flows, double-eddy circulations were observed, which causes concentrations in the middle inside of the street canyons and on the leeward of backwind buildings in all height ratios. Moreover, the concentration of pollutants will worsen for increases of building height perpendicular to the flow direction. The study also revealed PM_10_ dispersion in perpendicular and parallel flow under different height ratios and establishes that the worst pollutant situation is for higher upwind buildings for perpendicular flow. Within the meteorological conditions, this can support pre-warning mechanisms for prevention of PM_10_. Similarly, the study illustrates that large gaps (>15 m) will separate a long street canyon and form crossroad flow, which induce double-eddy circulations inside the street canyon and change the flow structure and the position of PM_10_ concentrations. Moreover, the result of the study confirms that there will be PM_10_ concentrations in the backwind buildings during perpendicular flows due to double-eddy circulations outside the street canyon, indicating that the measurements should be taken on both the inside and outside of the street canyon. Furthermore, this study reveals that the vertical influence of PM_10_ in those situations could be mitigated by designing effective government regulations. Preventive measures should be targeted at heights below 20 m, specifically below 4 m. Similarly, the positions of PM_10_ mass concentration in accordance with wind direction, street configuration, and size of gaps in adjacent buildings may be better mitigated by using dust shields, dust protection film, atomized water and ground wetting. Nevertheless, this needs to be backed up by government regulations on PM_10_ prevention derived from construction activities in a bid to reduce the exhaust emissions from heavy construction. Future research opportunities exist to examine the effects of different roughness indexes in various cities.

## Figures and Tables

**Table 1:** Classification of street canyons.

Caption	W (m)	L (m)	H (m)	H_A_ (m)	H_B_ (m)	H_A_/H_B_	Classification
model 1	30	355	15	15	30	0.5	Step-up street canyon
model 2	30	355	15	15	15	1	Symmetric street canyon
model 3	30	355	15	30	15	2	Step-down street canyon

**Table 2:** PM_10_ concentrations at 1.5 m height for measurement and numerical simulation in model 1.

PM_10_ Concentration (μg m^−3^)	Measurement			Numerical Simulation *X*_2_	Deviation |*X*_2_−*X*_1_|/*X*_1_
	Average Value *X*_1_	Max.	Min.		
Point 1	52	57	24	51	1.9%
Point 2	47	55	14	45	4.2%
Point 3	47	54	46	44	6.3%
Point 4	28	35	18	25	10.7%
Point 5	32	46	15	28	12.5%
Point 6	41	53	16	39	4.8%
Point 7	43	48	19	45	4.6%
Point 8	47	57	33	50	6.3%
Mean value	42	51	23	40	4.7%

**Table 3:** Mean values of PM_10_ in Z = 0.1H (μg m^−3^).

Caption	Parallel Flow	Perpendicular Flow	
	West Wind	North Wind	South Wind
model 1	18.7	18.6	20.2
model 2	17.8	21.8	19.8
model 3	17.7	27.2	17.6
